# Long-term and longitudinal nutrient stoichiometry changes in oligotrophic cascade reservoirs with trout cage aquaculture

**DOI:** 10.1038/s41598-020-68866-7

**Published:** 2020-08-10

**Authors:** Shiyu Miao, Shenglong Jian, Yang Liu, Changzhong Li, Hongtao Guan, Kemao Li, Guojie Wang, Zhenji Wang

**Affiliations:** 1 College of Eco-Environmental Engineering, Qinghai University, Xining, 810016 People’s Republic of China; 2 Qinghai Provincial Fishery Environmental Monitoring Center, Xining, 810012 Qinghai People’s Republic of China; 3 The Key Laboratory of Plateau Aquatic Organism and Ecological Environment in Qinghai, Qinghai Provincial Fishery Environmental Monitoring Center, Xining, 810012 Qinghai People’s Republic of China; 4 State Key Laboratory of Plateau Ecology and Agriculture, Qinghai University, Xining, 810016 People’s Republic of China

**Keywords:** Marine chemistry, Environmental chemistry, Environmental impact, Hydrology

## Abstract

The potential nutrient stoichiometry changes caused by trout cage aquaculture is concerned especially in oligotrophic waters. Long-term total nitrogen (N), total phosphorus (P) and N:P ratio changes in 6 cascade reservoirs with rainbow trout cage aquaculture in the oligotrophic upstream Yellow River (UYR) were studied from 2013 to 2017 in this paper. The 5-year monitoring results showed that N, P and N:P ratio levels showed no obvious long-term changes in high-altitude oligotrophic waters with rainbow trout cage aquaculture. No obvious longitudinal N, P and N:P ratio level changes were observed in the 6 cascade reservoirs from upstream Longyangxia Reservoir (LYR) to downstream Jishixia Reservoir (JSR). The increased N and P resulting from the cage aquaculture accounted only for 1.74% and 5.2% of the natural N and P levels, respectively, with a fish production of 10,000 tonnes. The upstream Yellow River remained oligotrophic and phosphorus-limited. Results in this study proved that trout cage aquaculture do not necessarily cause nitrogen, phosphorus and N:P ratio changes even in oligotrophic waters. Phosphorus should be considered first when identifying priority nitrogen and phosphorus sources and the corresponding control measures in waters with high N:P ratio.

## Introduction

Open-cage aquaculture relies on formulated feed for high-density aquaculture production, allows wastes, mainly uneaten food, faeces and urinary products, to be released directly into the surrounding water, and may cause significant impacts on a local scale, particularly in some concentrated production areas, such as shallow or confined water bodies^1–4^. There has been growing concern about the environmental impacts of open-cage aquaculture^5^, and obtaining sufficient knowledge of the environmental impacts of open-cage aquaculture has been encouraged for future developments in the aquaculture industry to ensure sustainability of the practice. Potential environmental impacts of open-cage aquaculture, such as genetic introgression of farmed salmonid in wild populations, regulatory effects of salmonid lice and viral diseases on wild salmonid populations, and the local and regional impact of nutrients and organic load and chemical usage^6–14^, have been identified and gradually understood. Nutrients discharged from cage aquaculture, especially phosphorus are among the most important concerns about the environmental impacts of cage aquaculture^15,16^. Mass balance models have been constructed to estimate the total and dissolved loss of nutrients to pelagic water environment and the accumulation of nutrients in sediments^1,17–19^. The amounts of nitrogen (N) and phosphorus (P) emissions to water environments during salmonid cage aquaculture are closely related to fish species, growth stage, feed quality, feeding methods and environmental conditions^6,10,20^. Abundant data indicate that the nutrients harvested in the form of fishery products during the caged salmonid farming process generally account for only approximately 30% of the total nutrients fed to the fish, and the rest are discharged into the environment as solid or dissolved aquaculture wastes^17,21^. Approximately 62% of the total nitrogen (TN) and 70% of the total phosphorus (TP) in feed were released into the environment in Norway^18^. The potential nutrient stoichiometry changes caused by salmon cage aquaculture in oligotrophic waters is especially concerned and of focus worldwide. However, little is known about the long-term nutrient stoichiometry changes in oligotrophic waters.

N and P are essential nutrient elements for phytoplankton growth, especially P, which is often the limiting factor for freshwater phytoplankton^22^. N and P from cage aquaculture not only increases the N and P concentrations, resulting in hypernutrition, but also changes the nitrogen to phosphorus ratio (N:P), which is often easily neglected. N and P enrichment by wastes from aquaculture cages was reported to stimulate the growth of algae, accelerate the eutrophication process and change the community structure and function of phytoplankton^17^, particularly in N-limited or P-limited oligotrophic aquatic systems^1,23–25^.

Previous studies have shown that the intensity and extent of the ecological and environmental impacts of the discharged nutrient wastes are related to the production scale, aquaculture method, water environment characteristics, biological community characteristics, hydrological characteristics, environmental management measures, etc.^6,10,24,26^. Existing literature about estimating the total N and P inputs and the environmental impacts of cage aquaculture are mostly focused on offshore areas^17^, lakes and specific reservoirs. Large rivers with cascade reservoirs often include lake-type reservoirs and river-type waterways. The high transparency, relatively high temperature and environment stable lake-type reservoirs was connected with river-type waterways with high water flow velocity and large sediment content. The migration and transformation of N and P varies in river-type waterways and lake-type reservoirs due to differences in water hydrodynamic and environment characteristics. Cascade reservoirs reduce the water flow velocity, trap suspended sediments and increase hydraulic retention time^27–29^, and alter the quantity and timing of N and P transported from upstream to downstream^30^. The nutrient stoichiometry changes caused by N and P discharge from aquaculture cages in large river with cascade reservoirs may be different with those in coastal waters, lakes and specific reservoirs due to the uniqueness and complexity of the hydrological characteristics. Presently, the knowledge about N, P and N:P ratio changes in cascade reservoirs over a long-term period and large spatial span were still lacking.

The Yellow River, originating in the Qinghai-Tibet Plateau, is the second-largest river in China. The upstream part of the Yellow River is located in the northeast of the Qinghai-Tibet Plateau. More than 10 large cascade reservoirs, such as Longyangxia Reservoir (LYR), Laxiwa Reservoir (LXR), Lijiaxia Reservoir (LJR), Gongboxia Reservoir (GBR), Suzhi Reservoir (SZR) and Jishixia Reservoir (JSR), have been built in the upstream Yellow River (UYR). The built cascade reservoirs, with a length of approximately 300 km and a drop of 800 m, provide unique natural cold water resources for trout farming. Since the beginning of the trout industry in 2009, the trout production scale has been on the rise, and in 2017, the production of trout, mainly triploid rainbow trout *Oncorhynchus mykiss* (Walbaum), reached 13,800 tonnes. Waters from LYR to JSR in UYR were oligotrophic and severe phosphorus limited^31^. Given the potential environmental impacts of cage aquaculture and the oligotrophic ecosystem in the upper Yellow River, local fisheries management departments have tried to decrease the impacts of cage aquaculture on the plateau river by adopting environmental monitoring and management measures, such as controlling the aquaculture production scale (30,000 tonnes), limiting the aquaculture density (6 kg/m^3^) and installing faecal collection devices. The changes of N, P and N:P ratio and the nutrient contribution of salmonid aquaculture in the high-elevation upstream Yellow River with cascade reservoirs had been monitored ever since.

In this paper, the long-term N, P and N:P ratio changes in the oligotrophic upstream Yellow River under the triploid rainbow trout aquaculture conditions from 2013 to 2017, and the effects of the cascade reservoirs longitudinal from LYR to JSR on N, P and N:P changes, were analysed. The results of this study would contribute to a better understanding of the long-term contributions of trout cage aquaculture to the nutrient stoichiometry changes in oligotrophic waters and the effects of cascade reservoirs to the nutrient stoichiometry.

## Materials and methods

### Study area and sampling locations

**Figure 1:**
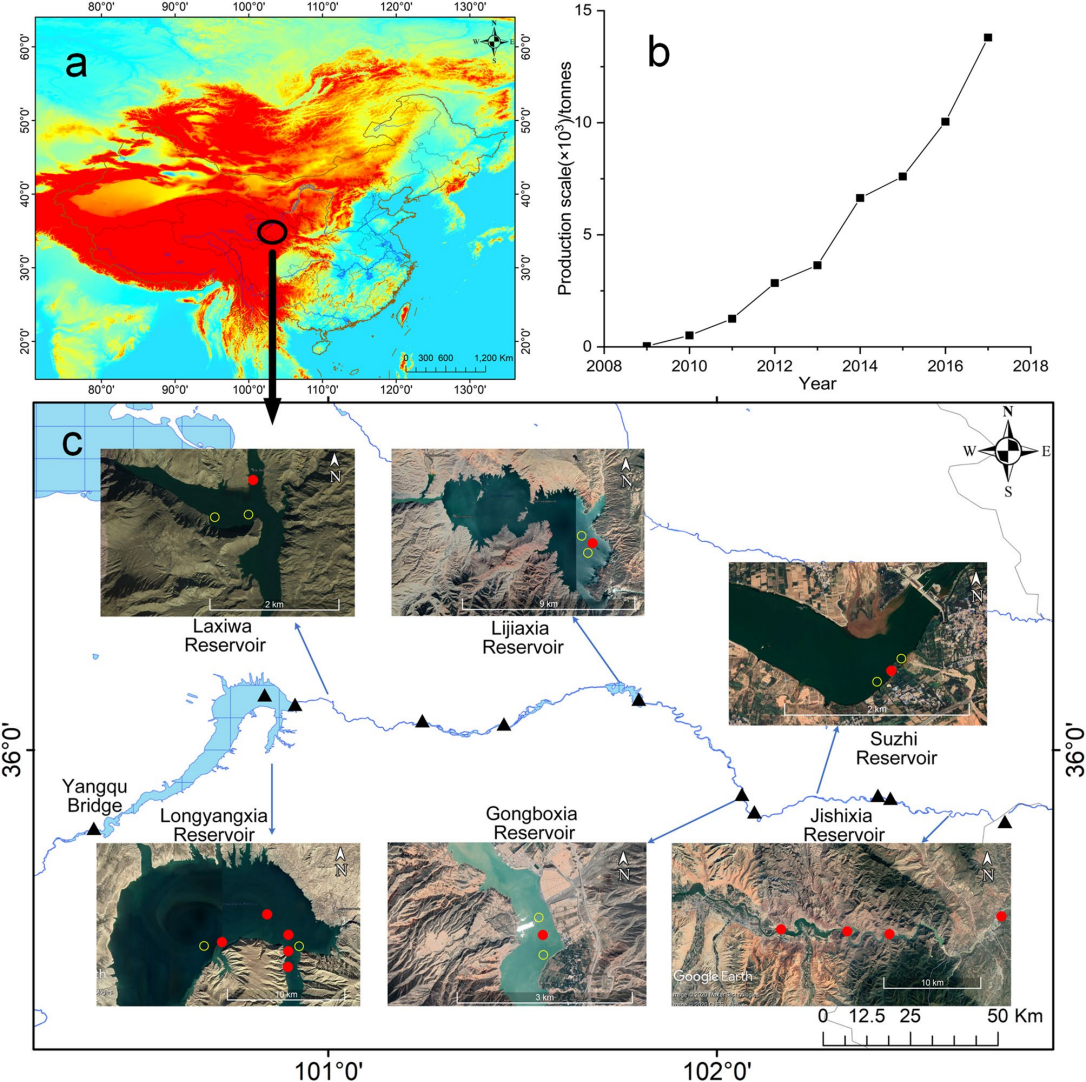
Schematic diagram of location of the cascade reservoirs (**a**) (Created by ArcGIS 10.5, www.esri.com), the trout production scale from 2009 to 2017 (**b**), and sampling points in the upstream Yellow River from LYR to JSR (**c**) (Created by ArcGIS 10.5, www.esri.com; Google Earth 7.3.3.7699, www.google.com/earth/). Solid red and hollow yellow circles indicated sampling points in CAA, with solid red circles indicating sampling points inside the cage groups, and hollow yellow circles indicating sampling points upstream or downstream the cages. Solid red circles also indicated where the trout cages groups located in UYR. Solid black triangle indicated sampling points in NAA.

The cage aquaculture area in this study is located in the upstream Yellow River from LYR to JSR, including LYR, LXR, LJR, GBR, SZR, JSR, in the northeastern Qinghai-Tibet Plateau, China (Fig. 1a). The average water depth for LYR, LXR, LJR, GBR, SZR and JSR is 64.0 m, 77.4 m, 51.6 m, 28.2 m, 6.7 m, and 19.4 m, respectively (Supplementary Table S1)^32^. Water environmental conditions in cascade reservoirs, such as LYR, LXR, LJR, GBR, SZR and JSR, are often stable with deep water, relatively slow water flow velocity, high transparency, and relatively high water temperature. Waterways between the aforementioned cascade reservoirs tend to have more rapid currents and higher sediment content. The cascade reservoirs studied in this research have cultured triploid rainbow trout *Oncorhynchus mykiss* (Walbaum) since 2009. The production scale has increased year by year, with the largest production scale reported in 2017, approximately 13,800 tonnes (Fig. 1b). The production scale for LYR, LXR, LJR, GBR, SZR and JSR was about 11,000 tonnes, 500 tonnes, 600 tonnes, 700 tonnes, 500 tonnes and 100 tonnes in 2017. Most culture cages are 15 m in depth, while some are 20 m in depth. The AAN level (60.2 ± 33.0 μmol/L) and AAP level (0.71 ± 0.34 μmol/L) in Yangqu Bridge (Fig. 1c), the main water inlet of LYR, from 2012 to 2017 was regarded as background level for N and P in the upstream Yellow River.

A total of 34 sampling points was set up both in non-aquaculture areas (NAA) in UYR and in cage aquaculture areas (CAA) in cascade reservoirs, including LYR, LXR, LJR, GBR, SZR and JSR. Of the 34 sampling points, 11 sampling points were set up in NAA, 7 sampling points were set up in LYR, 3 sampling points each were set up in LXR, LJR, GBR, SZR, respectively, and 4 sampling points were set up JSR (Fig. 1). Studies showed that near-field water column impacted by cage aquaculture were limited to only 20–50 metres^17,26^ and nutrient enrichment was not detectable beyond 100 m around the cages^24^. Sampling points in CAA were set up upstream (about 500 m away), inside the cage groups and downstream (about 500 m away), respectively. Sampling points in NAA were set up at least 2 km far from the aquaculture cages in UYR (Fig. 1). Sampling points in NAA were sampled at least twice a year, once in March or April and once in August or September. Sampling points in CAA were monitored once a quarter, 4 times throughout the year.

### Sample collection and determination

A glass vertical sampler was adopted to collect the water samples according to the Fishery Ecological Environment Monitoring Standard (SC/T 9102–2007, China). The sampling points were generally 1 m under the water surface. Three duplicates of total nitrogen (TN) and total phosphorus (TP) samples were collected. The samples were placed in a 100 mL polyethylene bottle immediately after collection, pre-treated with 3–4 drops of sulfuric acid (1:1), and stored at − 20 °C. TN was determined by alkaline potassium persulfate digestion and ultraviolet spectrophotometry (HJ 636–2012, China), and TP was determined by ammonium molybdate spectrophotometry (GB/T 11893–1989, China). N:P ratios (N:P) were calculated using the TN (μmol/L) and TP (μmol/L) concentrations. The N and P samples was collected and analysed by Qinghai Provincial Fishery Environmental Monitoring Center.

### Statistical analysis

Descriptive values of TN (μmol/L), TP (μmol/L) and N:P rations were expressed as the mean and standard deviation (mean ± s.d.). Statistical analysis was performed using SPSS software 20.0 (IBM, Armonk, NY, USA). One way analysis of variance (ANOVA) was performed to analyse the differences in the annual average N (AAN) concentrations, annual average P (AAP) concentrations and the annual average N:P ratio (AAR), respectively, from 2013 to 2017 in UYR, LYR, LXR, LJR, GBR, SZR, and JSR, respectively, and to analyse the differences in AAN, AAP and AAR, respectively, in the cascade reservoirs longitudinal from upstream LYR to downstream JSR in each year from 2013 to 2017. Homogeneity of variance was tested before ANOVA was performed, and if the test was passed, the LSD method was used, and if not, Tamhane's T2 method was used. Student's t-test was used to analyse the differences in the AAN, AAP, and AAR, respectively, between CAA and NAA in LYR, LXR, LJR, GBR, SZR, and JSR, respectively. Probabilities (P) of < 0.05 were considered significant (*), and probabilities (P) of < 0.01 were considered highly significant (**).

## Results

### Long-term nitrogen concentrations changes

**Figure 2:**
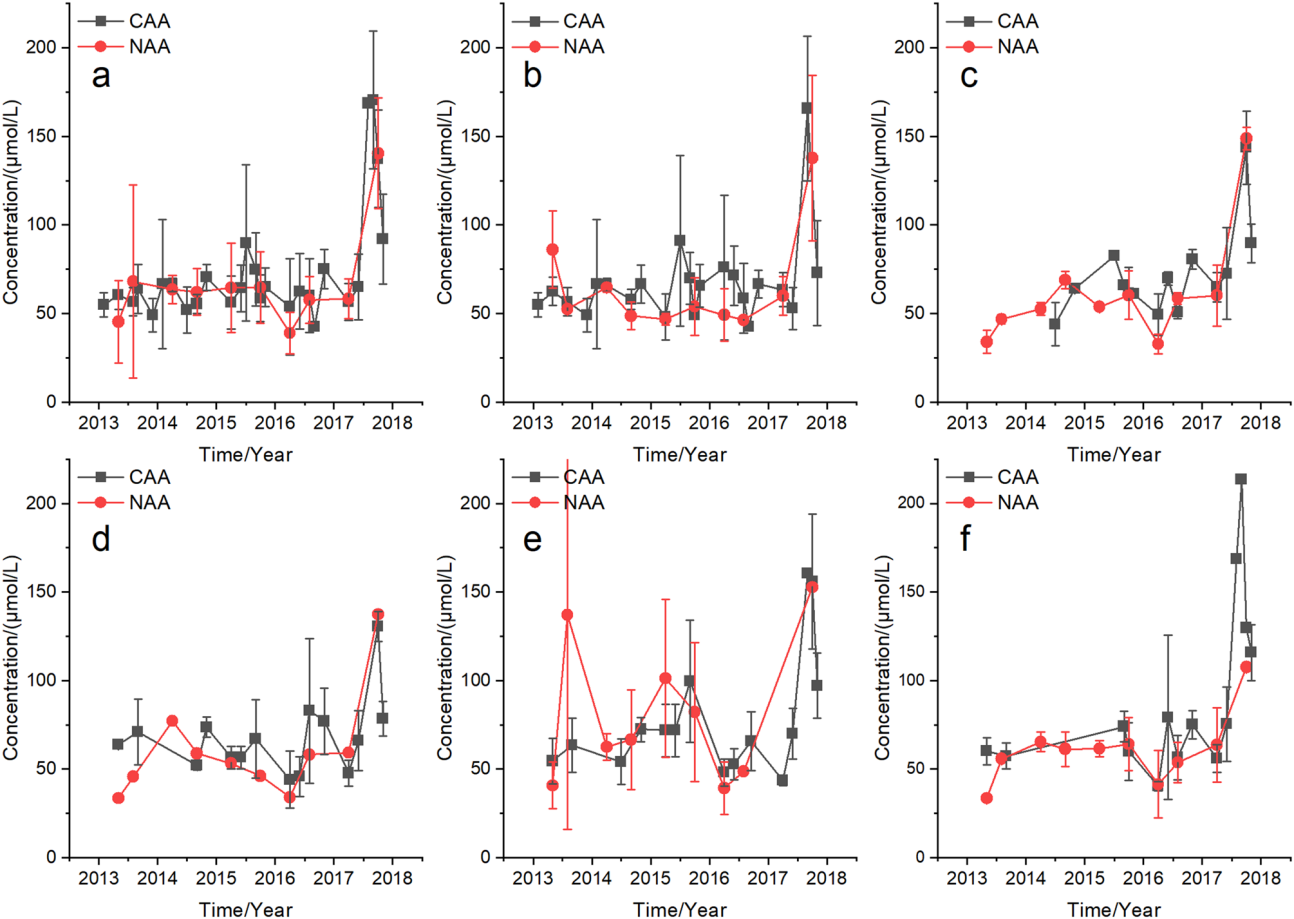
Quarterly average N concentrations (QAN) in non-aquaculture areas (NAA) and aquaculture areas (CAA) in the upstream Yellow River (UYR) from 2013 to 2017. (**a**) UYR, (**b**) LYR, (**c**) LXR, (**d**) LJR, (**e**) GBR, and (**f**) SZR.

Generally no obvious increase in the quarterly average N (QAN) concentrations (Fig. 2) and no significant difference in the AAN concentrations (Supplementary Table S2) in both NAA and CAA from 2013 to 2016 was observed in UYR (Fig. 2a), including LYR (Fig. 2b), LXR (Fig. 2c), LJR (Fig. 2d), GBR (Fig. 2e), and SZR (Fig. 2f), respectively. The QAN increased in the summer and autumn of 2017 in UYR in both CAA and NAA, but decreased to background level in winter of 2017 in CAA. No data was monitored in winter of 2017 in NAA (Fig. 2). The AAN concentrations in 2017 showed no significant differences with that in most years from 2013 to 2016 in both NAA and CAA in UYR, LYR, LXR, LJR, GBR, and SZR (CAA) (Supplementary Table S2).

Little differences of QAN between NAA and CAA were observed in each year from 2013 to 2017 in UYR, LYR, LXR, LJR, GBR, and SZR, respectively (Fig. 2). And except for a particular reservoir in a particular year, there were no significant differences in the AAN concentrations between CAA and NAA in UYR, LYR, LXR, LJR, GBR, and SZR (Supplementary Table S2).

### Long-term phosphorus concentrations changes

**Figure 3:**
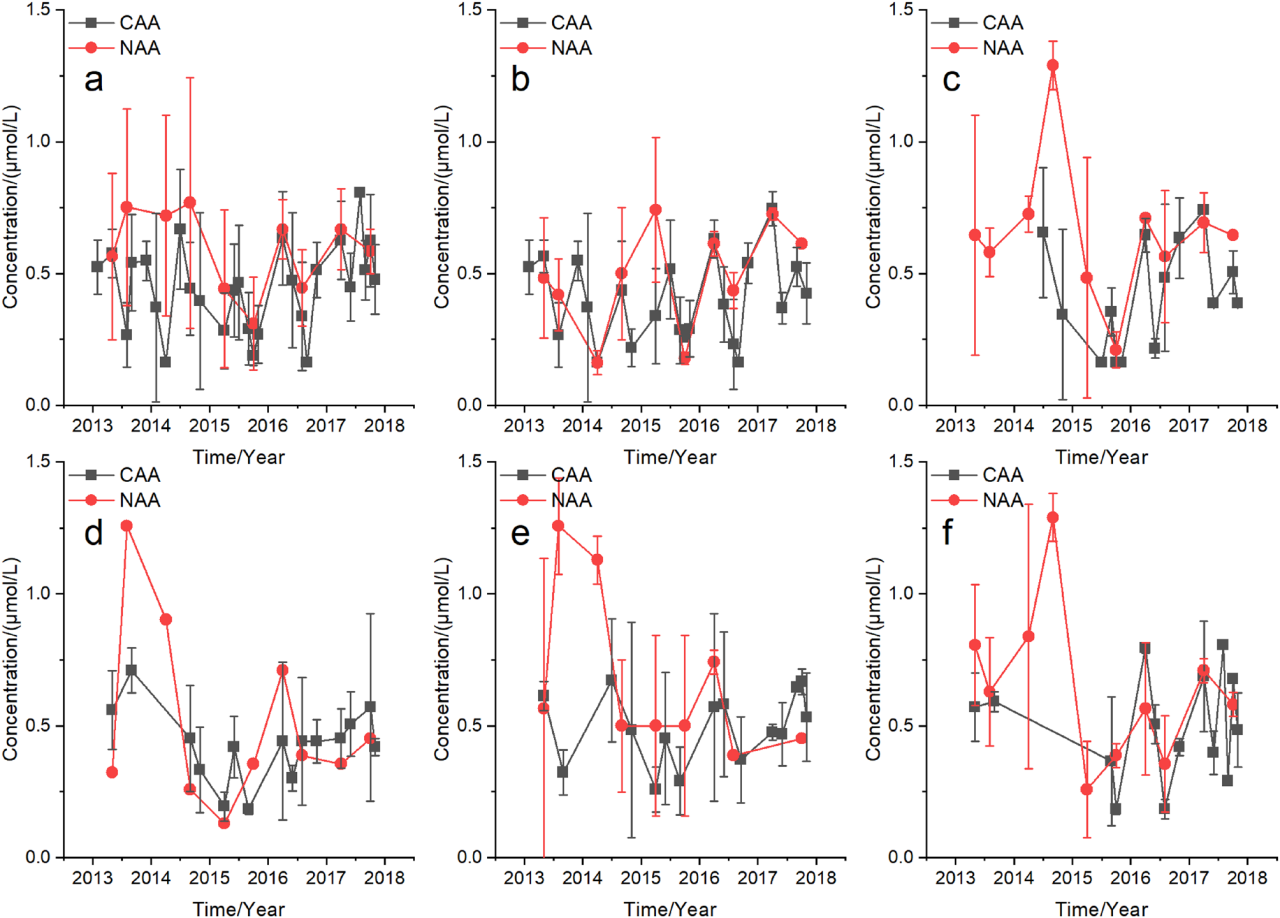
Quarterly average P (QAP) concentrations in non-aquaculture areas (NAA) and aquaculture areas (CAA) in the upper Yellow River (UYR) from 2013 to 2017. (**a**) UYR, (**b**) LYR, (**c**) LXR, (**d**) LJR, (**e**) GBR, and (**f**) SZR.

Generally the quarterly average P (QAP) concentrations fluctuated in both NAA and CAA from 2013 to 2017 in UYR (Fig. 3a), including LYR (Fig. 3b), LXR (Fig. 3c), LJR (Fig. 3d), GBR (Fig. 3e), and SZR (Fig. 3f), respectively. And the AAP concentrations remained relatively stable from 2013 to 2017, with no significant AAP concentration increases observed (Supplementary Table S3).

Differences in the QAP concentrations between NAA and CAA were not obvious in UYR, LYR, LXR, LJR, GBR, and SZR, respectively (Fig. 3). And no significant differences in the AAP concentrations between the CAA and NAA were observed in UYR, LYR, LXR, LJR, GBR, and SZR, respectively (Supplementary Table S3).

### Long-term N:P ratio changes

**Figure 4:**
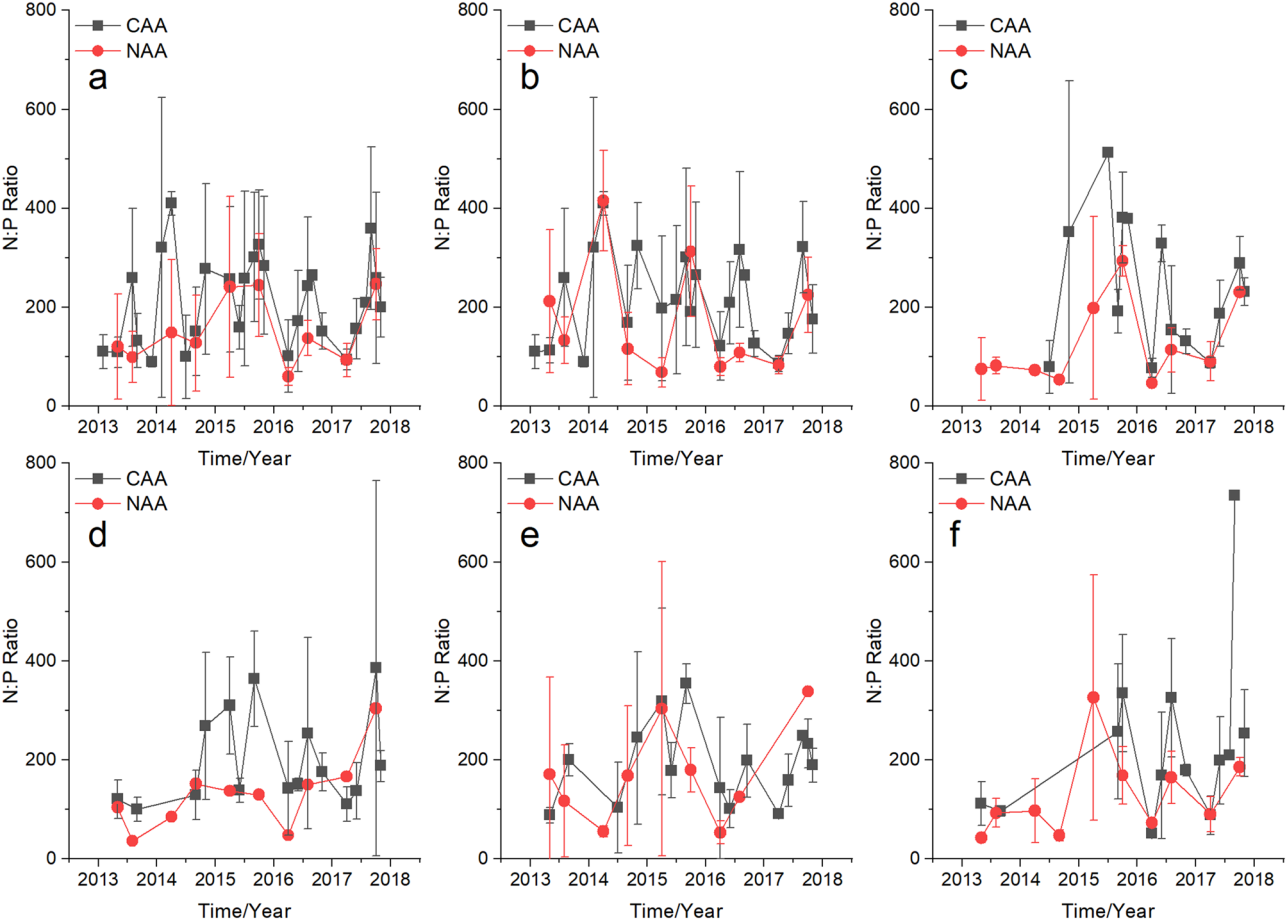
Quarterly average N:P ratio in non-aquaculture areas (NAA) and aquaculture areas (CAA) in the upstream Yellow River (UYR) from 2013 to 2017. (**a**) UYR, (**b**) LYR, (**c**) LXR, (**d**) LJR, (**e**) GBR, and (**f**) SZR.

Sampling results showed that the quarterly average N:P ratio (QAR) in UYR was significantly higher than the Redfield ratio (16:1) (Fig. 4), which indicated that UYR were significantly phosphorus limited. QAR results showed that the N:P ratios fluctuated at background levels from 2013 to 2017 in both NAA and CAA in UYR (Fig. 4a), including LYR (Fig. 4b), LXR (Fig. 4c), LJR (Fig. 4d), GBR (Fig. 4e), and SZR (Fig. 4f), respectively. And one-way analysis of variance (ANOVA) showed that the AAR generally fluctuated insignificantly in both NAA and CAA from 2013 to 2017 (Supplementary Table S4), indicating that the AAR generally did not increase or decrease with time in UYR, LYR, LXR, LJR, GBR, and SZR.

In most cases, no significant differences were observed for the AAR between the CAA and NAA in UYR, LYR, LXR, LJR, GBR, and SZR, respectively, in each year from 2013 to 2017 (Supplementary Table S4), indicating that the N:P ratio in CAA remained unchanged compared with that in the NAA.

### Longitudinal N, P and N:P changes in cascade reservoirs

**Figure 5:**
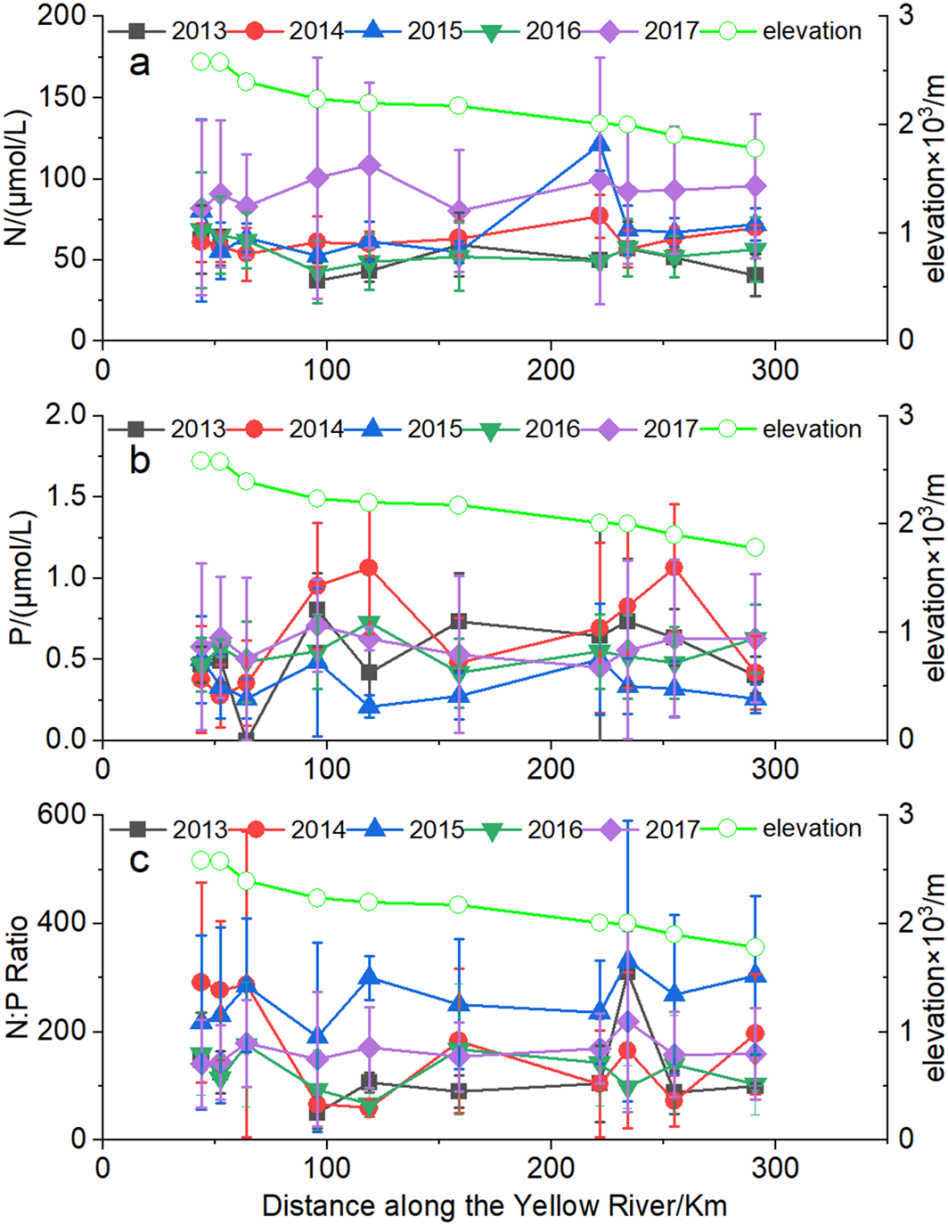
Annual average N (AAN, **a**), annual average P (AAP, **b**) and annual average N:P ratio (AAR, **c**) (left y-axis) and the elevation (right y-axis) changes longitudinally from LYR to JSR in the upstream Yellow River (UYR) from 2013 to 2017.

The elevation of cascade reservoirs from LYR to JSR in UYR decreases by 800 m (Fig. 5). The results showed that the AAN concentrations (Fig. 5a), AAP concentrations (Fig. 5b) and AAR (Fig. 5c), respectively, did not change obviously in cascade reservoirs longitudinal from the upstream LYR to the downstream JSR in UYR in each year from 2013 to 2017. And One-way analysis of variance (ANOVA) results showed that generally there were no significant changes in AAN, AAP, and AAR, respectively, in cascade reservoirs longitudinally from LYR to JSR in each year from 2013 to 2017 (Supplementary Table S5).

## Discussion

### N, P and N:P contributions of cage aquaculture

Our results in this study showed that the QAN concentrations (Fig. 2), QAP concentrations (Fig. 3), and QAR (Fig. 4), respectively, generally flocculated at natural levels and there were no significant differences in AAN concentrations (Supplementary Table S2), AAP concentrations (Supplementary Table S3) and AAR (Supplementary Table S4), respectively, between the CAA and NAA in UYR, LYR, LXR, LJR, GBR, and SZR, respectively, from 2013 to 2017. The unchanged N, P and N:P was due to the small production scale, large water flow, high water exchange rate, deep water and a series of environmental protection and management measures, including aquaculture regional planning, aquaculture carrying capacity estimation, aquaculture production scale control, and faeces collection in UYR. This research joined numerous studies that cage aquaculture showed no significant and long-term nutrient enrichment in aquaculture waters with good management and control measures, high water flow, high water exchange rate, and deep water^17,33,34,35^.

Phosphorus was a limiting nutrient element necessary for phytoplankton growth and reproduction in cascade reservoirs in UYR^31^. The Dillon-Rigler model was used to estimate the aquaculture carrying capacity of the reservoirs based on 10% of the remaining phosphorus emission capacity by the water quality standard (GB3838-2002) in UYR^32^. The maximum aquaculture carrying capacity in UYR was determined to be 30,000 tonnes, of which LYR 20,000 tonnes, and the remaining reservoirs 10,000 tonnes^32^. The planned net cage aquaculture areas only accounted for 0.1% of the total water surface areas in UYR. Apart from the aquaculture production scale, aquaculture density in UYR was stipulated to be lower than 6 kg/m^3^, much lower than 8–30 kg/m^3^, a density reported in salmonid cage aquaculture practices in some European countries. Faecal collectors were also installed at the bottom of the aquaculture cages to reduce the N and P discharge, and a special device was designed to concentrate and separate faeces from water. A recommended purchase list of environmentally friendly feed has been established to decrease the discharge of nutrients. The environmental management measures adopted in UYR decreased the discharged amount of N and P.

Rainbow trout aquaculture facilities, technology, feed and eyed eggs in the upper Yellow River were generally introduced from European countries. Of the total feed input, 62% of N and 70% of P was estimated to release into the environment in Norwegian salmon farms^18^. The content of crude protein, crude fat, crude ash, crude fiber, N and P in the feed used was about 40%, 28%, 7.5%, 3.0%, 7.2% and 1.2%, respectively. The total nutrient discharges were estimated with the feed N content (dry weight) of 7.2%^18,36^, feed P content (dry weight) of 1.2%^18,37^, and feed conversion rate (FCR) of 1.1^36^. The total N discharged into the environment per tonne of trout production was estimated to be approximately 49.1 kg, of which dissolved N was approximately 35.6 kg, and the total P was estimated to be approximately 9.24 kg, of which the excreted dissolved inorganic P and returned sedimentary P was approximately 2.11 kg. From 2013 to 2017, the production scale of trout increased gradually in the upstream Yellow River, with the highest production in 2017 (Fig. 1b). In 2017, approximately 51.2 × 10^4^ kg N and 7.7 × 10^4^ kg P were discharged into the environment, of which the dissolved N was approximately 35.6 × 10^4^ kg and the dissolved P was approximately 2.11 × 10^4^ kg. The average runoff in UYR was approximately 220 × 10^8^ m^3^/year. The increased N and P concentrations in UYR caused by cage aquaculture were estimated to be 16.2 μg/L and 0.096 μg/L, respectively, accounting for 1.74% and 5.2% of the natural N levels (Fig. 2) and P levels (Fig. 3), respectively in UYR. This result indicated that N and P discharges from cage aquaculture contributed little to the N and P levels and cage aquaculture was not the main source of N and P in UYR. The average N levels and average P levels from 2012 to 2019 in Yangqu Bridge, the main water inlet of LYR, was 60.2 ± 33.0 μmol/L and 0.71 ± 0.34 μmol/L, respectively. N and P in cascade reservoirs from LYR to SZR mainly sourced from the upper parts of Yellow River.

The UYR from LYR to JSR were oligotrophic, with low N and P concentrations (Fig. 2, 3), a high N:P ratio (Fig. 4) and diatom dominance^31^. There are worries that wastes from trout aquaculture cages may contribute to changed water nutrient stoichiometry since the N:P ratio in the wastes discharged by cage aquaculture was estimated to be about 11.8:1, significantly lower than that of UYR (Fig. 4). However, despite these concerns, we were pleased to see that from 2013 to 2017, the N:P ratio generally remained relatively unchanged at background levels and trout cage aquaculture, as well as other N and P pollution sources from watersheds, such as animal husbandry and domestic sewage from the villages, showed no significant long-term effects on the N:P ratios of the cascade reservoirs in UYR (Supplementary Table S1–S4). It is noteworthy that the upstream Yellow River was still oligotrophic and phosphorus-limited.

### Longitudinal N, P and N:P changes in cascade reservoirs

Our results showed that the N concentrations, P concentrations and N:P ratio fluctuated but generally remained relatively stable at background levels (Fig. 5) and no significant longitudinal changes for N concentrations, P concentrations and N:P ratio, respectively, in cascade reservoirs from the upstream LYR to the downstream SZR in UYR in each year from 2013 to 2017 were observed (Supplementary Table S5). The N concentrations, P concentrations and N:P ratio in cascade reservoirs depended on reservoir characteristics, such as size, residence time, location, ambient environment, anthropogenic pressures, etc*.*^38,39^. The average flow in the UYR is 680 m^3^/s and 220 × 10^8^ m^3^/year. The total water storage capacity of LYR, LXR, LJR, GBR, SZR and JSR is 247 × 10^8^ m^3^, 10.79 × 10^8^ m^3^, 16.5 × 10^8^ m^3^, 6.2 × 10^8^ m^3^, 0.455 × 10^8^ m^3^ and 2.64 × 10^8^ m^3^, respectively, and the drainage rate is 0.943 times/year, 19.28 times/year, 12.96 times/year, 36.45 times/year, 487.20 times/year and 83.71 times/year, respectively^32^. The results that no significant longitudinal changes of N, P and N:P observed in cascade reservoirs from the upstream LYR to the downstream SZR may be due to the small aquaculture production scale, large water flow and high water exchange rates in the downstream reservoirs. This results also highlighted the role of water flushing and diffusion in dispersing the N and P wastes discharged by the open aquaculture cages in the upstream Yellow River.

### Implications in N and P waste reduction in UYR

The exogenous N and P sources in UYR include soil and water loss, cage aquaculture, agro-pastoral activities as well other anthropic activities along the Yellow River. Diverse nitrogen and phosphorus sources have made it difficult to impose regulations on aquaculture nutrient discharge from cage operations, if not accompanied by parallel measures to decrease the influx due to land-based pollution^40^. Although there have been many research papers involving N and P discharges from open culture cages and their potential environmental impacts^17^, there is limited literature on establishing causality between increased N and P concentrations and cage aquaculture. It is not easy to identify causal links, especially as there are extensive sources of N and P in cage aquaculture waters, not only N and P inputs from cage aquaculture but also discharges from domestic sewage, industrial sewage, agriculture, etc. It is widely recognized that, in many aquaculture waters, nutrient inputs from agro-pastoral land have far exceeded those from aquaculture cages^17^. Approximately 50% of N in European cage aquaculture areas is loaded from large European rivers^17^. In Scottish aquaculture areas, an average of 80.5% of N and 44.4% of P was derived from agricultural and natural erosion, 12.6% of N and 36.6% of P was derived from urban waste, 0.8% of N and 5.1% of P was derived from industrial inputs, and only 5.45% of N and 12.6% of P was derived from salmon aquaculture inputs^41^. Globally, the relative contribution of aquaculture to N and P discharge is small^40^. The results in this study showed that N and P discharges from cage aquaculture contributed only 1.74% and 5.2% to the N and P levels, respectively. The N and P in UYR was supposed to be mainly derived from non-point sourced animal husbandry, agricultural and natural erosion since the catchment areas of UYR is sparsely populated. At present, the accurate total amounts and proportions of non-point sourced and point sourced N and P emissions in UYR are still not yet clear. An exogenous N and P pollutant source analysis and the identification of priority control sources in UYR are needed to put forward corresponding regulatory measures.

The UYR was still oligotrophic and significantly phosphorus-limited with an average N:P ratio above 200 (Fig. 4). From the perspective of N:P ratios and the phosphorus limitation of phytoplankton in UYR, to decrease the impacts of potential changes in the N:P ratio on the structure and function of aquatic ecosystems in UYR, water quality management in the upstream Yellow River and environmental management of salmonid cage aquaculture should focus on reducing the phosphorus loads.

## Conclusions

The potential nutrient stoichiometry changes caused by salmon cage aquaculture in oligotrophic waters is concerned worldwide. Triploid rainbow trout was cage cultured in high-elevation oligotrophic cascade reservoirs in the upstream Yellow River in Qinghai-Tibet Plateau. The 5-year and 300-km sampling results in our study showed that the N, P and N:P ratio generally changed insignificantly in the upstream Yellow River, including LYR, LXR, LJR, GBR, SZR and JSR, respectively, from 2013 to 2017, and no longitudinal changes in N, P and N:P ratio levels were observed in cascade reservoirs from LYR to JSR in each year from 2013 to 2017. The N and P concentrations increases resulting from the cage aquaculture accounted for only 1.74% and 5.2% of the natural N and P levels, respectively, with a production of 10,000 tonnes. The upstream Yellow River remained oligotrophic and phosphorus-limited. The minor nutrient enrichment from cage aquaculture was believed to be the result of relatively small production scale, high water flow, high water exchange rate and environmental protection and management measures in the upstream Yellow River. The results in this study proved that trout cage aquaculture do not necessarily cause nitrogen, phosphorus and N:P ratio changes even in oligotrophic waters. Analysis also showed that P should be considered first when identifying priority N and P sources and the corresponding control measures in waters with high N:P ratio.

## Supplementary information

Supplementary file1 (DOC 90 kb)
