# Supplementary material for: Long-term and longitudinal nutrient stoichiometry changes in oligotrophic cascade reservoirs with trout cage aquaculture
Source: Sci Rep. 2020 Aug 10;10:13483. doi: 10.1038/s41598-020-68866-7 (PMC7417551; doi:10.1038/s41598-020-68866-7)
Supplement: Supplementary file 1 — Supplementary file1 (DOC 90 kb) [file 41598_2020_68866_MOESM1_ESM.doc]

Long-term and longitudinal nutrient stoichiometry changes in oligotrophic cascade reservoirs with trout cage aquaculture

Shiyu Miao^1,a^, Shenglong Jian^2,3,a^, Yang Liu^1,4,*^, Changzhong Li^1,4^, Hongtao Guan^2,3^, Kemao Li^2,3^, Guojie Wang^2,3^, Zhenji Wang^2,3^

^1^College of Eco-Environmental Engineering, Qinghai University, Xining, 810016, People’s Republic of China

^2^Qinghai Provincial Fishery Environmental Monitoring Center, Xining, Qinghai, 810012, Peoples’ Republic of China

^3^The Key Laboratory of Plateau Aquatic Organism and Ecological Environment in Qinghai, Qinghai Provincial Fishery Environmental Monitoring Center, Xining, Qinghai, 810012, People’s Republic of China

^4^State Key Laboratory of Plateau Ecology and Agriculture, Qinghai University, Xining, 810016, People’s Republic of China

^a^ Shared first authorship.

^*^Corresponding author. E-mail: ecosea@outlook.com (Y. Liu). Tel: +86 971 531 0086.

Supplementary Table S1 Hydrodynamic characteristics of the cascade reservoirs in UYR

|  | LYR | LXR | LJR | GBR | SZR | JSR |
| --- | --- | --- | --- | --- | --- | --- |
| Area (×10^6^ m^2^) | 383 | 13.94 | 32 | 22 | 6.72 | 13.6 |
| Volume (×10^8^ m^3^) | 247 | 10.79 | 16.5 | 6.2 | 0.455 | 2.64 |
| Depth (m) | 64 | 77.4 | 51.6 | 28.2 | 6.7 | 19.41 |
| Runoff (×10^8^ m^3^) | 232.9 | 208 | 213.8 | 226 | 221.7 | 221 |
| Drainage rate (times year^–1^) | 0.943 | 19.28 | 12.96 | 36.45 | 487.2 | 83.71 |

Supplementary Table S2 Annual average N (AAN, μmol/L) in CAA and NAA in cascade reservoirs

| Year | UYR | | LYR | | LXR | | LJR | | GBR | | SZR | |
| --- | --- | --- | --- | --- | --- | --- | --- | --- | --- | --- | --- | --- |
|  | CAA | NAA | CAA | NAA | CAA | NAA | CAA | NAA | CAA | NAA | CAA | NAA |
| 2013 | 58.9±10.5^b^ | 56.7±42.4^b^ | 55.8±8.79^bB^ | 69.3±23.1 |  | 40.4±8.5^b^ | 67.4±12.4 | 39.6±8.6^bB^ | 59.1±13.6^*^ | 88.9±89.8 | 58.8±6.9^*^ | 44.6±12.9 |
| 2014 | 62.5±19.3^b^ | 62.9±10.6^b^ | 65±17.6^abA^ | 56.7±10.3 | 54.0±13.5^b^ | 60.7±10.1^b^ | 62.9±12.5 | 68.0±12.9ab^AB^ | 63.2±13.7 | 67.9±17 |  | 63.3±6.9 |
| 2015 | 68.6±23.8^b^ | 67.1±22.2^ab^ | 68±29.6^abA^ | 53.6±10.4 | 66.4±10.6^ab^ | 57.1±8.8^b^ | 60.1±13.1 | 49.7±5.1b^AB^ | 81.1±24.5 | 91.7±38.6 | 67.0±14.0 | 62.9±9.1 |
| 2016 | 62.7±26.4^b^ | 48.4±15.4^b^ | 67.6±24.1^abA^ | 47.9±8.7 | 62.6±15.0^ab^ | 45.6±15.3^b^ | 62.5±27.9 | 46.2±17.0^bB^ | 58.1±17.9 | 54.3±25.6 | 62.9±26.4 | 47.6±17.9 |
| 2017 | 91.1±44.1^a^ | 99.4±47.9^a^ | 88.7±52.1^aA^ | 98.8±52.8 | 92.7±35.6^a^ | 104.6±52.1^a^ | 80.7±33.6 | 98.4±55.3^aA^ | 91.9±46.9 | 99.3±52.9 | 104.4±55.7 | 85.7±28.1 |

Notes: Student's t-test was used to test the differences of AAN concentrations between CAA and the NAA with the significant differences denoted by ^*^ and the highly significant differences by ^**^. One way analysis of variance (ANOVA) was performed to analyse the differences in AAN concentrations in LYR, LXR, LJR, GBR, SZR and JSR, respectively, from 2013 to 2017 with the significant differences noted with lowercase letters and highly significant with capital letters. Statistical analysis was performed using SPSS software 20.0.

Supplementary Table S3 Annual average P (AAP, μmol/L) in CAA and NAA in cascade reservoirs

| Year | UYR | | LYR | | LXR | | LJR | | GBR | | SZR | |
| --- | --- | --- | --- | --- | --- | --- | --- | --- | --- | --- | --- | --- |
|  | CAA | NAA | CAA | NAA | CAA | NAA | CAA | NAA | CAA | NAA | CAA | NAA |
| 2013 | 0.52±0.14^a^ | 0.66±0.35^b^ | 0.48±0.15^aAB^ | 0.45±0.16 |  | 0.61±0.27^bA^ | 0.63±0.14^aA**^ | 0.79±0.66 | 0.47±0.17^ab^ | 0.91±0.53 | 0.58±0.09^a^ | 0.72±0.20^abAB^ |
| 2014 | 0.46±0.31^a^ | 0.74±0.42^a^ | 0.30±0.22^bC^ | 0.33±0.24 | 0.50±0.31^abA^ | 1.01±0.33^aA^ | 0.39±0.18^bcABC^ | 0.58±0.46 | 0.58±0.34^a^ | 0.81±0.39 |  | 1.06±0.39^aA^ |
| 2015 | 0.31±0.16^b^ | 0.38±0.25^a^ | 0.36±0.17^abBC*^ | 0.46±0.36 | 0.23±0.11^bB^ | 0.35±0.31^bB^ | 0.27±0.13^cC^ | 0.24±0.16 | 0.33±0.17^b^ | 0.50±0.28 | 0.27±0.18^b^ | 0.32±0.13^cB^ |
| 2016 | 0.49±0.22^a^ | 0.56±0.17^b^ | 0.44±0.19^aABC^ | 0.52±0.11 | 0.49±0.23^aA^ | 0.64±0.17^abA^ | 0.41±0.18^bcBC^ | 0.55±0.23 | 0.47±0.25^ab^ | 0.62±0.17 | 0.48±0.23^a^ | 0.46±0.22^bcB^ |
| 2017 | 0.53±0.16^a^ | 0.63±0.13^b^ | 0.52±0.17^aA^ | 0.67±0.07 | 0.51±0.16^aA^ | 0.67±0.07^abA^ | 0.49±0.18^abAB^ | 0.40±0.07 | 0.53±0.12^ab^ | 0.60±0.18 | 0.54±0.20^a^ | 0.65±0.08^bcAB^ |

Notes: Student's t-test was used to test the differences of the AAP concentrations between CAA and the NAA with the significant differences denoted by ^*^ and the highly significant differences by ^**^. One way analysis of variance (ANOVA) was performed to analyse the differences in the AAP concentrations in LYR, LXR, LJR, GBR, SZR and JSR, respectively, from 2013 to 2017 with the significant differences noted with lowercase letters and highly significant with capital letters. Statistical analysis was performed using SPSS software 20.0.

Supplementary Table S4 Annual average N:P Ratio (AAR) in CAA and NAA in cascade reservoirs

| Year | UYR | | LYR | | LXR | | LJR | | GBR | | SZR | |
| --- | --- | --- | --- | --- | --- | --- | --- | --- | --- | --- | --- | --- |
|  | CAA | NAA | CAA | NAA | CAA | NAA | CAA | NAA | CAA | NAA | CAA | NAA |
| 2013 | 130.8±73.5^cB^ | 109.1±82.2^bAB^ | 143.0±95.8 | 172.4±99.1 |  | 78.4±38.0 | 110.7±31.1^b^ | 70.2±47.9^c^ | 144.5±65.3^*^ | 144.2±134.5 | 104.8±30.1^b^ | 68.2±33.9 |
| 2014 | 222.4±174.8^abAB^ | 138.0±122.0^abAB^ | 305.6±175.5 | 265.5±187.4 | 215.5±246.5^ab^ | 63.1±11.7 | 199.2±125.1^ab^ | 156.7±100.9^abc^ | 174.6±154.0 | 112.3±104.5 |  | 72.6±47.5 |
| 2015 | 270.6±136.0^aA^ | 242.8±145.0^aA^ | 241.7±148.6 | 190.5±161.0 | 339.4±133.3^a^ | 245.6±121.2 | 271.1±123.4^a^ | 271.4±200.2^a^ | 284.1±128.7 | 241.9±187.8 | 296.2±121.8^a^ | 247.8±173.1 |
| 2016 | 166.9±106.2bcB^**^ | 98.5±48.4^bB^ | 191.2±115.9 | 93.9±22.4 | 172.6±114.6^b*^ | 79.9±46.9 | 181.0±104.0^ab^ | 99.3±72.3^bc^ | 160.9±91.1 | 94.9±50.2 | 181.3±126.3^ab^ | 119.1±61.2 |
| 2017 | 186.0±119.6^bB^ | 170.0±95.9^abAB^ | 182.6±105.6 | 153.7±93.5 | 198.8±86.2^ab^ | 160.4±84.5 | 205.6±199.5^ab^ | 235.6±97.2^ab^ | 169.3±64.1 | 169.4±121.2 | 230.1±179.9^ab^ | 138.5±59.6 |

Notes: Student's t-test was used to test the difference in the AAR between CAA and the NAA, with the significant differences shown by ^*^, and very significant differences shown by ^**^. One-way analysis of variance (ANOVA) was performed to analyse the differences in AAR of LYR, LXR, LJR, GBR and SZR from 2013 to 2017, with significant differences shown by lowercase letters and very significant differences shown by capital letters. Statistical analysis was performed using SPSS software 20.0.

Supplementary Table S5 Longitudinal AAN (μmol/L), AAP (μmol/L) and AAR changes in cascade reservoirs from LYR to JSR.

| Distance | Site | 2013 | | | 2014 | | | 2015 | | | 2016 | | | 2017 | | |
| --- | --- | --- | --- | --- | --- | --- | --- | --- | --- | --- | --- | --- | --- | --- | --- | --- |
| (km) |  | TN | TP | N:P | TN | TP | N:P | TN | TP | N:P | TN | TP | N:P | TN | TP | N:P |
| 0-53 | LYR | 60.1±16.3^a^ | 0.48±0.13 | 136.6±66.2^a^ | 59.6±10.1 | 0.32±0.26 | 284.3±151.9^a^ | 68.0±41.6^a^ | 0.42±0.23 | 224.1±154.1 | 67.0±32.1 | 0.52±0.13^a^ | 137.3±62.6 | 86.6±47.6 | 0.61±0.13 | 142.7±71.9 |
| 64-96 | LXR | 37.1±7.9^b^ | 0.81±0.16 | 50.0±27.9^b^ | 56.2±20.7 | 0.55±0.42 | 214.0±247.4^ab^ | 52.5±9.1^a^ | 0.32±0.26 | 254.6±133.3 | 58.1±18.4 | 0.48±0.23^a^ | 160.0±110.8 | 86.9±38.2 | 0.55±0.16 | 173.5±82.8 |
| 119-158 | LJR | 55.6±18.6^a^ | 0.65±0.29 | 94.0±27.7^ab^ | 62.6±9.7 | 0.61±0.39 | 152.6±127.0^ab^ | 56.5±11.4^a^ | 0.26±0.13 | 261.2±109.4 | 51.6±19.8 | 0.48±0.23^b^ | 150.6±116.6 | 85.1±38.9 | 0.55±0.16 | 157.2±62.4 |
| 221-234 | GBR | 56.0±15.6^a^ | 0.71±0.42 | 117.4±104.7^ab^ | 61.9±14.3 | 0.81±0.48 | 119.5±104.0^b^ | 83.6±26.5^a^ | 0.35±0.19 | 255.5±126.7 | 56.1±16.2 | 0.52±0.26^a^ | 135.6±74.7 | 98.6±46.3 | 0.55±0.16 | 177.6±79.9 |
| 234-255 | SZR | 51.5±12.1^a^ | 0.65±0.16 | 88.9±39.3^ab^ | 61.9±6.9 | 1.06±0.39 | 72.6±47.5^b^ | 66.9±8.9^a^ | 0.32±0.16 | 269.5±146.8 | 51.9±12.6 | 0.48±0.23^a^ | 140.2±90.6 | 95.7±39.1 | 0.65±0.16 | 158.3±78.8 |
| 255-291 | JSR | 40.7±9.3^a^ | 0.42±0.10 | 100.4±4.2^ab^ | 69.8±1.2 | 0.42±0.16 | 196.6±111.0^ab^ | 72.0±7.1^b^ | 0.26±0.06 | 304.8±146.7 | 56.6±19.7 | 0.65±0.23^a^ | 103.7±57.0 | 95.6±44.5 | 0.65±0.13 | 159.6±84.1 |

Notes: One-way analysis of variance (ANOVA) was performed to analyse the differences in AAN, AAP, and AAR, respectively, from upstream LYR to downstream JSR in each year from 2013 to 2017, with significant differences shown by lowercase letters and very significant differences shown by capital letters. Statistical analysis was performed using SPSS software 20.0.
